# Long‐Term Patient‐Reported Outcomes and Dosimetric Predictors of Quality of Life After Curative‐Intent Radiation Therapy for Oropharyngeal Squamous Cell Carcinoma: A Cross‐Sectional Study

**DOI:** 10.1002/cnr2.70712

**Published:** 2026-10-07

**Authors:** Anna Lawless, Dasantha Jayamanne, Lisa Parker, Esther Duruchukwu, Paula Macleod, Venkatesha Venkatesha, Christopher Brown, Alexander Guminski, Adrian Lee, Michael Back, Thomas Eade, Sarah Bergamin

**Affiliations:** ^1^ Northern Sydney Clinical School, Faculty of Medicine and Health The University of Sydney Sydney New South Wales Australia; ^2^ Department of Radiation Oncology Northern Sydney Cancer Centre, Royal North Shore Hospital St Leonards New South Wales Australia; ^3^ Northern Sydney Local Health District, Royal North Shore Hospital St Leonards New South Wales Australia; ^4^ Department of Medical Oncology Northern Sydney Cancer Centre, Royal North Shore Hospital St Leonards New South Wales Australia

**Keywords:** head and neck, oropharyngeal cancer, radiation therapy, squamous cell carcinoma

## Abstract

**Background:**

Radiation therapy (RT) is central to curative treatment for oropharyngeal squamous cell carcinoma (OPSCC) but may cause late side effects that can adversely impact quality of life (QOL). As OPSCC treatment becomes more personalised, clinically meaningful QOL endpoints and their predictors are increasingly important.

**Aims:**

To assess long‐term QOL, treatment regret, and predictors of QOL after curative‐intent RT for OPSCC.

**Methods and Results:**

This cross‐sectional survey included patients with primary OPSCC treated with curative intent RT between 2007 and 2023 at a single tertiary institution. Patients completed validated EORTC QOL questionnaires and additional items assessing perceived health and treatment regret. Survival outcomes were estimated using Kaplan–Meier methods, and compared by p16 status using log‐rank test. QOL scores were analysed as continuous variables using univariate and multivariable linear regression models, with prespecified dosimetric predictors tested against clinically corresponding EORTC QOL‐HN43 symptom scales and adjusted for multiple comparisons. Of the 344 patients treated, 274 (80%) had p16‐positive disease. At median follow of 7.7 years, 5‐year overall survival was 90% for p16‐positive versus 52% for p16‐negative disease, and disease‐free survival 89% versus 72%. Of 232 patients contacted, 186 (80%) completed the QOL survey at median 6.3 years after RT. 173 (93%) reported no treatment regret regarding RT. Mean global health status was 81.3/100, while dry mouth/sticky saliva was the most affected domain (mean 41.9/100). On multivariable analysis, older age was associated with worse global health status (−4.9 points per 10 years, *p* = 0.001) and higher contralateral mean parotoid dose with worse dry mouth/sticky saliva scores (10.1 points per 10 Gy, 95% CI: 3.7 to 16.5, *p* = 0.002). Chemotherapy and prior surgery were not associated with any domain.

**Conclusion:**

Long‐term survivors of OPSCC reported favourable global QOL and little treatment regret after RT. However, dry mouth/sticky saliva remained a substantial burden, and more likely with higher contralateral parotid mean dose. These findings support ongoing contralateral parotid sparing and consideration of unilateral neck irradiation when oncologically appropriate, while recognising the cross‐sectional cohort and need for rigorous prospective studies in this area.

## 1 Introduction

The incidence and survival rates of human papillomavirus associated oropharyngeal squamous cell carcinoma (HPV‐OPSCC) are steadily increasing in high income countries [1, 2, 3, 4, 5]. Despite the effectiveness of the HPV vaccine, this trend can be expected to continue for several decades due to the long latency between infection and development of malignancy and low worldwide vaccination rates [6]. Thus, numbers of long‐term survivors of this disease with treatment‐related side effects can be expected to continue to increase, with growing survivorship needs. There is a need to focus on how to better understand and optimise long‐term quality of life (QOL).

Despite improvements in RT techniques over the past decades (intensity modulated and image‐guided radiotherapy, IMRT and IGRT respectively) and increasing understanding of optimal dose constraints to specific organs at risk (e.g., Parotid, pharyngeal constrictors) to reduce toxicities and/or improve QOL [7, 8, 9], the acute and late treatment‐related toxicities of RT can still be considerable. Permanent late treatment‐related side effects are reported in majority of patients treated for head and neck cancers and can significantly impact patients' QOL [10]. These late effects can include xerostomia, dysphagia, dysgeusia, dental problems, trismus, neck fibrosis, lymphedema and altered speech [11]. For patients with oropharyngeal cancers specifically (as compared to infrahyoidal or early larynx cancers), toxicity profiles are different, with more severe symptom scores and greater rates of severe dysphagia [12]. Surgery followed by adjuvant (chemo)radiotherapy and definitive (chemo)radiotherapy appear to demonstrate broadly comparable, albeit modality‐specific toxicity and quality of life profiles, although the highest symptom burden, in particular dysphagia, is associated with trimodality treatment [13].

Despite the interest in different approaches to treatment de‐escalation, many patients with HPV‐OPSCC (as well as HPV‐negative OPSCC) will continue to receive standard chemoradiotherapy, and thus identifying dosimetric predictors of late QOL is important to further refine and improve treatments for patients continuing to receive standard high dose treatment. Understanding and quantifying the late QOL outcomes associated with standard treatment is crucial to provide a baseline comparison to the oncological outcomes in trial cohorts. This study aimed to report real world oncological and late QOL results of a large cohort of patients with OPSCC treated at a single tertiary institution over almost two decades, and to identify predictors of worse QOL. Ultimately, we aimed to inform future RT protocol refinements and better tailor survivorship care.

## 2 Materials and Methods

Patients with biopsy confirmed primary OPSCC who received curative intent RT, with or without prior surgery, between January 2007 and May 2023 at a large urban teaching hospital comprised the study population. Patients were identified and their data extracted from a prospectively maintained institutional database and corroborated with information from the electronic medical record where required.

All patients underwent diagnostic FDG‐PET/CT and were discussed at a dedicated Head and Neck Multidisciplinary meeting prior to treatment. For patients undergoing primary surgical management, adjuvant RT was recommended for adverse pathologic features such as one or more of: extranodal extension (ENE), positive or close (≤ 2 mm) margins on the primary tumour, single lymph node greater than 3 cm, multiple positive lymph nodes, nodal disease in levels IV or V, perineural invasion, or lymphovascular invasion, as per current international guidelines [14, 15]. Concurrent chemotherapy (cisplatin 40 mg/m^2^ weekly unless contraindicated) was typically recommended for all patients undergoing definitive RT except those with node‐negative disease and small primary tumours (T1‐2N0). For patients undergoing adjuvant RT, concurrent chemotherapy was recommended in the presence of ENE or involved surgical margins.

RT was delivered via IMRT or volumetric modulated arc therapy (VMAT) technique using pre‐operative FDG‐PET/CT fusion, a thermoplastic head and neck mask for immobilisation, and daily cone beam CT for image verification. In the definitive setting, RT was delivered to a total dose of 70Gy in 35 fractions over 7 weeks, with three dose levels (70, 63 and 56 Gy to the high, intermediate and low risk clinical treatment volumes (CTV)), as per previously published guidelines [16, 17, 18]. The contralateral neck was spared for well‐lateralised primary tonsil cancers (> 1 cm from midline) without involvement of the soft palate or base of tongue. In the adjuvant setting, RT was delivered to the surgical bed and at‐risk lymph node areas to a dose of 60Gy in 30 fractions delivered 5 times per week over 6 weeks, with a concomitant boost to 63Gy to any areas with involved margins. All treatment plans were generated using the Eclipse treatment planning system (Varian Medical Systems, Palo Alto, CA, USA). Dosimetric parameters were extracted and checked by a head and neck radiation oncologist (RO). Where pre‐specified OARs of interest were missing, they were retrospectively contoured by a single head and neck RO using standardised nomenclature and current departmental contouring guidelines.

All patients who were alive in late 2023 were contacted by telephone and invited to participate in the QOL survey. Those who verbally agreed were sent the survey and up to two reminders. The survey included all 43 items from the European Organization for Research and Treatment of Cancer (EORTC) Quality of Life Questionnaire Head and Neck Module 43 (QLQ HN‐43) [19], the two global health assessment items from the QLQ‐C30, and additional questions regarding perceived health and treatment regret (see Figure S1 for entire survey as distributed to participants). For brevity, only items 29 and 30 of the EORTC QLQ‐C30, which together comprise the global health status/QOL scale, were administered. The QLQ‐C30 global health status/QOL scale and QLQ‐HN43 symptom scales were scored according to official EORTC scoring procedures, with raw scores linearly transformed to a 0–100 scale. Higher QLQ‐HN43 scores indicate greater symptom burden (worse QOL), whereas higher QLQ‐C30 global health status/QOL scores indicate better overall health and QOL.

Clinical and pathologic information was extracted from a prospective institutional database. Recurrence, date of last follow up, and survival status were corroborated with clinical follow up records, imaging, and pathology reports from the electronic medical record where applicable. Descriptive statistics were used to summarise patient and treatment characteristics. Kaplan–Meier curves were used to calculate survival outcomes (calculated from the date of starting RT). The Chi‐square test of independence or Fisher's exact test to account for sparse data was used to test associations between categorical variables. The threshold for statistical significance for the two‐sided *p*‐values was set at *p* < 0.05.

All QOL outcomes were analysed as continuous variables scored according to the official EORTC scoring manuals and linearly transformed to 0 to 100 scale. Five outcomes were pre‐specified: QLQ‐C30 global health status scale, four QLQ‐HN43 symptom scales selected for their clinical relevance and its dosimetry (swallowing, dry mouth and sticky saliva, speech, and pain in the mouth). Associations with clinicopathological factors were analysed using linear regression model based on heteroscedasticity consistent robust (HC3) standard errors and reported as univariate and multivariable adjusted mean differences (or slope) with 95% CI. The p16 status and ECOG status could not be included in the multivariable model due to lack of variation with only 3 patients (out of 186) being p16‐negative and 94% were ECOG 0. Dosimetric analyses were restricted to 11 prespecified organ‐at‐risk or target‐volume and outcome pairs rather than testing all dosimetric variables against all domains: mean contralateral parotid, ipsilateral parotid and oral cavity dose against dry mouth and sticky saliva; mean pharyngeal constrictor and larynx dose against swallowing; mean larynx and oral cavity dose against speech; mean oral cavity dose against pain in the mouth; and CTV high, intermediate and low dose volumes against global health status.

Dose parameters were modelled per 10 Gy and volumes per 10 cc, unadjusted first and then adjusted for age, smoking, stage, chemotherapy and time since RT, with Benjamini‐Hochberg false‐discovery‐rate (FDR) *q*‐values reported across the family of 11 comparisons. OS and DFS were compared between p16‐positive and p16‐negative patients using the log‐rank test and univariable Cox proportional hazards models.

The QOL analyses included the 186 patients who completed the survey, among whom all questionnaire items, model covariates and dosimetric parameters were complete, so no imputation analysis was required; the extent of missing data in the full cohort and a comparison of survey completers with non‐completers are provided in Table S2. As a retrospective cohort study including all eligible patients treated during the study period, no a priori sample size or power calculation was performed and 95% CI are reported throughout. Analyses were performed in R, version 4.5.3.

## 3 Results

344 patients underwent RT for primary OPSCC during the study period. Demographic information and treatment details are summarised in Table 1. Median age was 61 years, 298 patients (87%) were male, and 274 patients (80%) had p16‐positive tumours. 19 patients (6%) had undergone resection of the primary tumour as well as neck dissection prior to RT. Prescribed RT dose was 70Gy in 35 fractions in 290 patients (84%). RT encompassed the primary site in 342 patients (99%) and bilateral neck in 291 patients (85%). 290 patients (84%) received chemotherapy, delivered concurrently in 286 patients (98% of those that received chemotherapy). 117 patients (34%) had an inpatient admission during or within 6 weeks of completing RT. Among the 186 survey completers with dosimetric data available, median high‐, intermediate‐ and low‐dose CTV volumes were 62 cc (IQR 41–87), 67 cc (IQR 49–98) and 130 cc (IQR 87–171) respectively. Median combined pharyngeal‐constrictor, oral cavity, ipsilateral parotid, and contralateral parotid mean doses were 55Gy (IQR 50–59), oral cavity 46Gy (IQR 39–51), 29Gy (IQR 23–42) and 19Gy (IQR 10–24) respectively.

**TABLE 1:** Patient, tumour and treatment characteristics for patients treated with radiation therapy for oropharyngeal squamous cell carcinoma.

Characteristic	*N* = 344^a^
Age at starting RT	61 (55, 67)
Sex	
Female	46 (13%)
Male	298 (87%)
ECOG	
0	299 (89%)
≥ 1	36 (11%)
Unknown	9
Smoking	
Current	56 (16%)
Ex‐Smoker	160 (47%)
Non‐Smoker	127 (37%)
Unknown	1
Alcohol	
Yes	277 (83%)
Unknown	10
Subsite	
Tonsil	196 (57%)
Base of tongue	130 (38%)
Other	18 (5.2%)
p16	
Positive	274 (80%)
Negative	26 (7.6%)
Not tested	44 (13%)
Staging (AJCC 8th edition)	
Stage I	141 (41%)
Stage II	118 (34%)
Stage III	43 (13%)
Stage IVA	40 (12%)
Stage IVB	2 (0.6%)
Prior surgery	
Definitive surgical resection	19 (6%)
Prior diagnostic surgical procedure	41 (12%)
No	285 (83%)
Primary site treated with RT	
Yes	342 (99%)
Neck treated with RT	
Unilateral	53 (15%)
Bilateral	291 (85%)
Chemotherapy	
Any	290 (84%)
Neoadjuvant	36 (12%)
Concurrent	286 (98%)
RT Prescribed Dose	
70Gy/35#	290 (84%)
66Gy/30#	31 (9.0%)
63Gy/30#	8 (2.3%)
Other	15 (4.4%)
RT Intent	
Definitive	326 (95%)
Adjuvant	18 (5.2%)
PEG insertion	
Yes	271 (79%)
Unknown	1
Admission during or within 6 weeks of completing RT	
Yes	117 (34%)
Unknown	1
RT course completed in full	
Yes	341 (99%)
Dosimetric data (*N* = 186)^b^	
GTVprimary (volume, cc)	8 (3, 14)
GTVnode (volume, cc)	9 (2, 18)
CTV high dose (volume, cc)	62 (41, 87)
CTV intermediate dose (volume, cc)	67 (49, 98)
CTV low dose (volume, cc)	130 (87, 171)
Ipsilateral parotid mean dose (Gy)	29 (23, 42)
Contralateral parotid mean dose (Gy)	19 (10, 24)
Oral cavity mean dose (Gy)	46 (39, 51)
Superior pharyngeal constrictor mean dose (Gy)	60 (52, 64)
Middle pharyngeal constrictor mean dose (Gy)	59 (53, 63)
Inferior pharyngeal constrictor mean dose (Gy)	46 (7, 52)
Pharyngeal constrictors (combined) mean dose (Gy)	55 (50, 59)
Larynx mean dose (Gy)	32 (25, 35)

Abbreviation: RT, radiation therapy.

^a^ Median (IQR); *n* (%).

^b^ Dosimetric data for patients who completed quality of life survey (*N* = 186).

Median follow up was 7.7 years, during which time 84 patients died. Estimated 1‐ and 5‐year OS were 96% and 85%, and median OS was 15.0 years (Figure 1A). Estimated 1‐ and 5‐year DFS were 93% and 81%, and median DFS was 15.4 years (Figure 2A). OS and DFS were significantly longer for patients with p16‐positive disease compared to p16‐negative disease (5‐year OS 90% vs. 52%, 5y DFS 89% vs. 72%, both log‐rank *p* < 0.001, Figures 1B and 2B). Of the 54 patients who experienced disease recurrence during the study period; 22 experienced isolated locoregional failure (LRF), 13 LRF with concurrent distant metastases, and 19 distant‐only metastases. Eight patients developed new head and neck (including cutaneous) cancers during follow up. Among patients with known p16 status, recurrence occurred in 13/26 (50%) p16‐negative and 35/274 (13%) p16‐positive cases (Table 2). Isolated LRF occurred in 8 (31%) versus 14 (5%), LRF with distant failure in 2 (8%) versus 9 (3%), and distant‐only failure in 3 (12%) versus 12 (4%) respectively. Both any recurrence and the overall pattern of first failure differed by p16 status (both *p* < 0.001).

**FIGURE 1:**
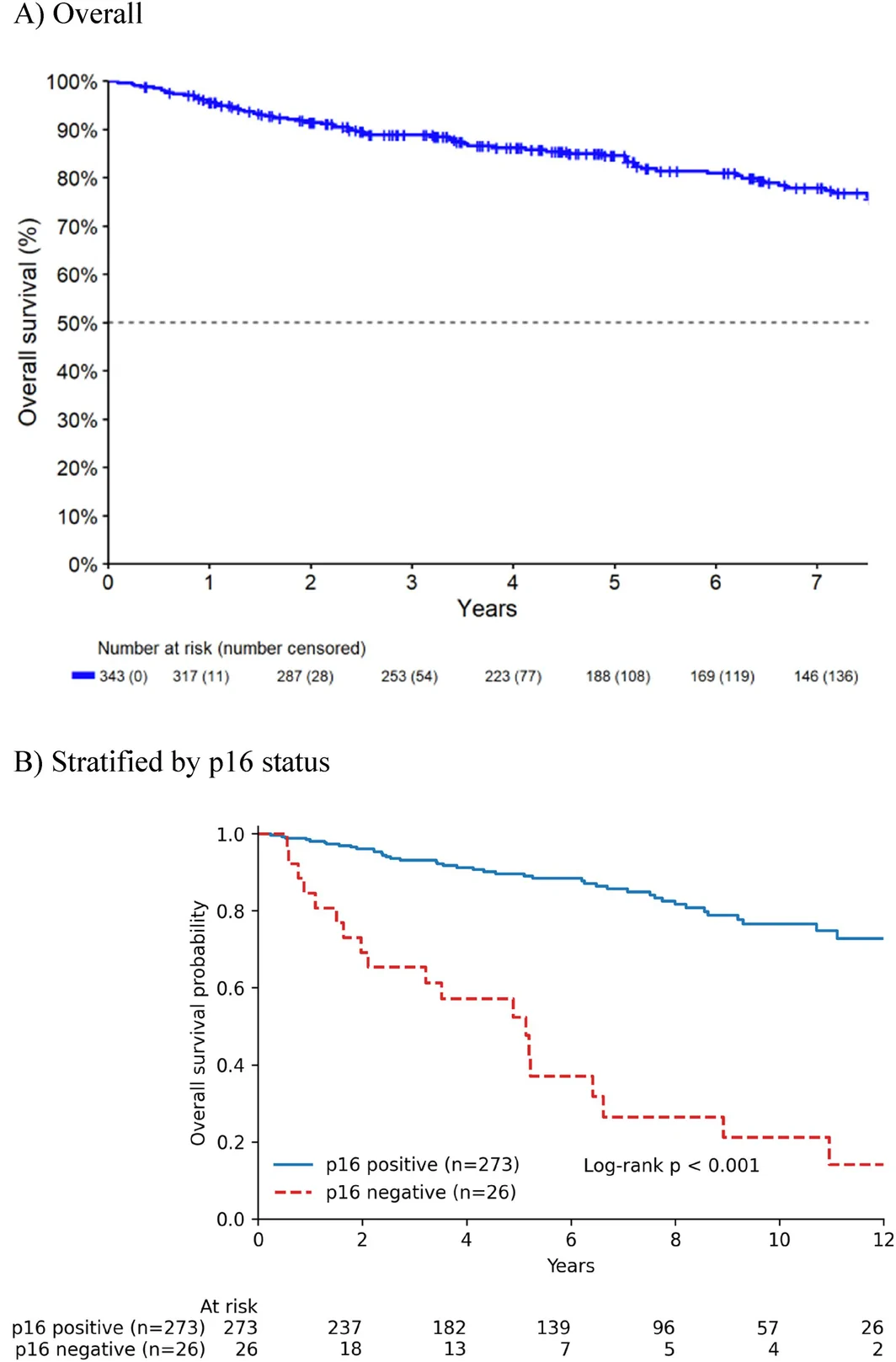
Kaplan–Meier estimate of overall survival in patients with oropharyngeal squamous cell carcinoma treated with radiation therapy, both overall (1A) and stratified by p16 status (1B). One p‐16 positive patient was excluded from survival analyses owing to a missing follow‐up date.

**FIGURE 2:**
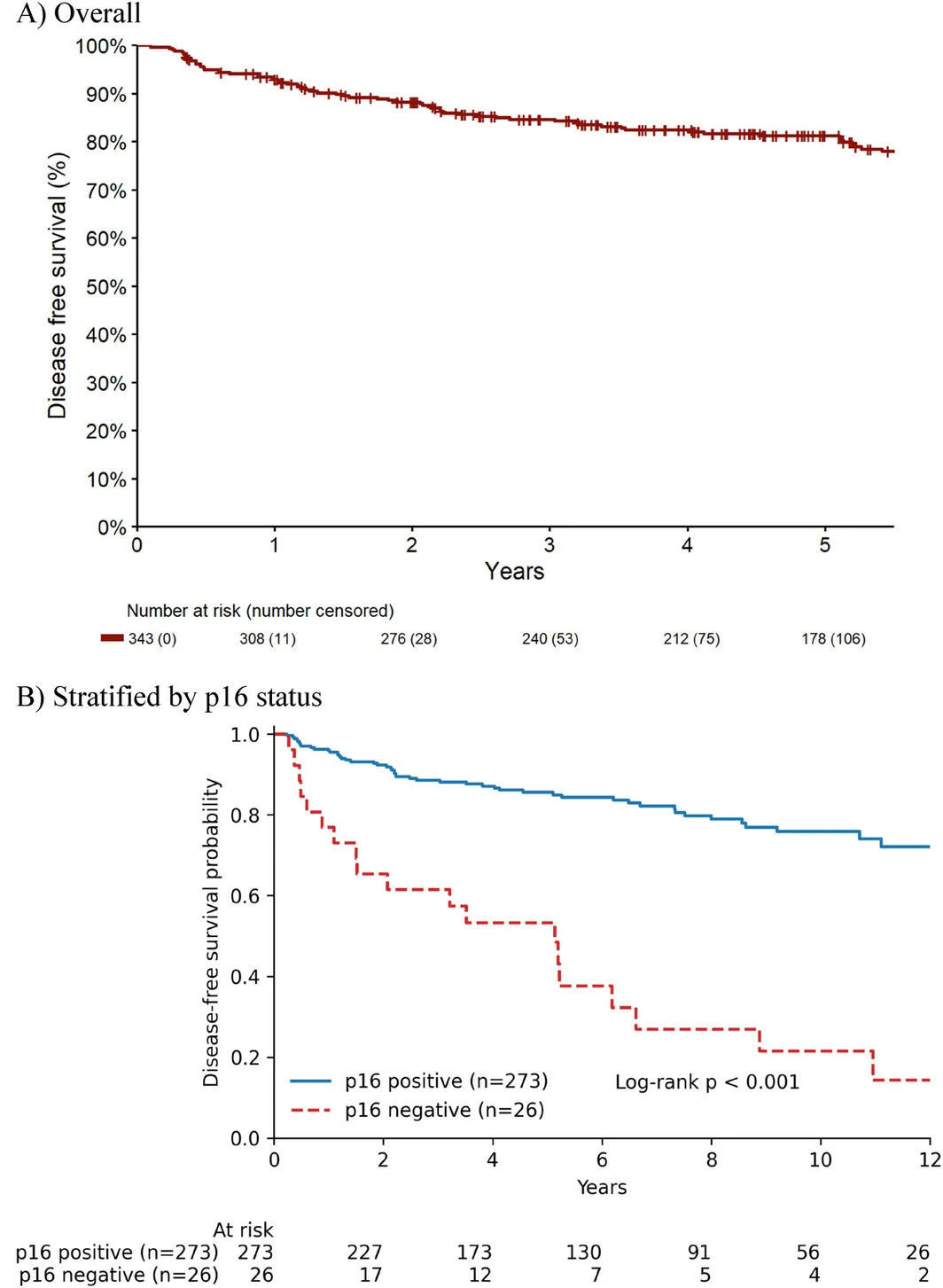
Kaplan–Meier estimate of disease‐free survival in patients with oropharyngeal squamous cell carcinoma treated with radiation therapy, overall (2A) and stratified by p16 status (2B). One p‐16 positive patient was excluded from survival analyses owing to a missing follow‐up date.

**TABLE 2:** Patterns of first failure by p16 status.

Pattern of failure	p16‐negative (*n* = 26)	p16‐positive (*n* = 273)
No recurrence	13 (50%)	239 (88%)
Isolated LRF	8 (31%)	14 (5%)
LRF + distant	2 (8%)	9 (3%)
Distant only	3 (12%)	12 (4%)

*Note:* Percentages are within each column (p16 group).

232 participants were contacted by telephone and invited to complete the QOL survey (Figure S1), of which 186 patients (80%) completed the survey in full. 95 patients were not contacted (82 deceased, 13 for other reasons), and 17 patients could not be reached via available contact details (Figure S2). The median time from completion of RT to completion of the survey was 6.3 years (IQR 3.3–10.5). Detailed and synthesised survey responses are shown in Table 3, with additional information in Table S3. 30 patients (16%) reported severe side effects since RT. Compared to their health before RT, 51 patients described their health as ‘somewhat worse’ (24%) or ‘much worse’ (3.8%), of which 36/51 patients (71%) attributed this worsening to RT. Overall, 173 patients (93%) reported no treatment regret regarding receipt of RT.

**TABLE 3:** Responses to the quality of life survey in patients with oropharyngeal squamous cell carcinoma treated with radiation therapy.

Characteristic	*N* = 186^a^
Current ECOG performance status	
ECOG 0	143 (77%)
ECOG 1	33 (18%)
ECOG 2	6 (3.2%)
ECOG 3	2 (1.1%)
ECOG 4	1 (0.5%)
Other	1 (0.5%)
Severe side effects since RT (e.g., Side effects requiring major surgery, hyperbaric oxygen and/or hospitalisation)	30 (16%)
No	156 (84%)
Self‐reported health (compared to before RT)	
Much better	21 (11%)
Somewhat better	23 (12%)
About the same	88 (47%)
Somewhat worse	44 (24%)
Much worse	7 (3.8%)
Unsure	3 (1.6%)
If ‘somewhat worse’ or ‘much worse’, do you think this is due to RT?	
Yes	36 (63%)
No	15 (29%)
Treatment Regret	
Not at all	173 (93%)
A little	8 (4.3%)
Quite a lot	3 (1.6%)
Very much	2 (1.1%)

^a^ *n* (%); Median (IQR).

Calculated EORTC QLQ‐C30 (global health status) and EORTC HN‐43 scores are shown in Table 4, and survey responses for EORTC HN‐43 in Table S1. Median global health status score was 83.3/100 (IQR 66.7, 100). Among QLQ HN‐43 symptom scales, dry mouth/sticky saliva was most affected (median 33.3, IQR 16.7–66.7; mean 41.9). Domains with a median score of 0/100 (least affected) included body image, sexuality, shoulder or skin problems, mouth opening, coughing, social contact, neck swelling, weight loss, wound healing problems, and neurological problems.

**TABLE 4:** EORTC QLQ‐C30 global health status and QLQ‐HN‐43 symptom‐scale scores in patients with oropharyngeal squamous cell carcinoma treated with radiation therapy.

Characteristic	*N* = 186	
	Median (IQR)	Mean (minimum–maximum)
EORTC QLQ‐30		
Global Health Status	83.3 (66.7, 100)	81.3 (16.7–100)
EORTC HN‐43		
Pain in the head and neck	8.3 (0, 16.7)	12.3 (0–83.3)
Problems with swallowing	16.7 (0, 25)	19 (0–83.3)
Problems with teeth	11.1 (0–44.4)	25 (0–100)
Dry mouth & sticky saliva	33.3 (16.7, 66.7)	41.9 (0–100)
Problems with senses	16.7 (0, 33.3)	22.9 (0–100)
Speech problems	6.7 (0, 20)	15.6 (0–100)
Body image	0 (0, 22.2)	13 (0–100)
Social eating	8.3 (0, 25)	20.6 (0–100)
Problems with sexuality	0 (0, 33.3)	19.1 (0–100)
Shoulder problems	0 (0, 16.7)	12.7 (0–100)
Skin problems	0 (0, 22.2)	9.9 (0–66.7)
Anxiety	16.7 (0, 33.3)	23 (0–100)
Problems opening mouth	0 (0, 33.3)	20.4 (0–100)
Coughing	0 (0, 33.3)	19.5 (0–100)
Problems with social contact	0 (0, 0)	5.6 (0–100)
Neck swelling	0 (0, 0)	10.8 (0–100)
Weight loss	0 (0, 0)	10.2 (0–100)
Wound healing problems	0 (0, 0)	8.8 (0–100)
Neurological problems	0 (0, 33.3)	21.3 (0–100)

Results of the linear regression for predictors of continuous QOL scores are reported in Table 5. On multivariable analysis, older age was associated with worse global health status (−4.9 points per 10 years, 95% CI −7.8 to −1.9, *p* = 0.001) and worse swallowing scores (3.7 points per 10 years, 95% CI 0.2–7.2, *p* = 0.037). Current smoking showed a possible association with worse global health status compared with never smokers, approaching statistical significance, although the confidence interval crossed the null (−10.9 points, 95% CI −22.5 to 0.6, *p* = 0.063). Unilateral neck irradiation was associated with lower dry mouth and sticky saliva scores than bilateral irradiation (−14.0 points, 95% CI −27.6 to −0.5, *p* = 0.042). Neither prior surgery nor chemotherapy was associated with any of the five QOL outcomes. Longer time since RT was associated with marginally worse speech scores (1.0 point per year, 95% CI 0.03–2.0, *p* = 0.043). Among the 11 prespecified dosimetric comparisons (Table 6), higher mean contralateral parotid dose was associated with worse dry mouth and sticky saliva scores (10.1 points per 10 Gy, 95% CI 3.7–16.5, *p* = 0.002), which remained significant after Benjamini‐Hochberg FDR adjustment (*q* = 0.021). Ipsilateral parotid dose showed a weaker association in the same direction (5.0 points per 10 Gy, 95% CI 0.4–9.6, *p* = 0.034, *q* = 0.186). The association between CTV intermediate dose volume and global health status was attenuated and no longer significant after adjustment (−0.6 points per 10 cc, 95% CI −1.2 to 0.04, *p* = 0.064, *q* = 0.235), and no association was observed for CTV low dose volume. Pharyngeal constrictor and larynx doses were not associated with swallowing or speech scores.

**TABLE 5:** Prognostic factors for global quality of life (per EORTC QLQ 30 global health status) and head and neck specific quality of life (per EORTC HN‐43) for patients with oropharyngeal squamous cell carcinoma treated with radiation therapy.

Predictor	Univariable *β* (95% CI)	*p*	Multivariable *β* (95% CI)	*p*
QLQ‐C30 Global health status (higher = better QOL)				
Age at RT (per 10 y)	−3.8 (−6.8 to −0.8)	0.013	−4.9 (−7.8 to −1.9)	0.001
Smoking: former vs. never	−1.2 (−6.7 to 4.3)	0.671	−1.3 (−6.9 to 4.3)	0.653
Smoking: current vs. never	−11.1 (−22.4 to 0.1)	0.052	−10.9 (−22.5 to 0.6)	0.063
Chemotherapy: yes vs. no	5.4 (−3.1 to 13.9)	0.211	4.7 (−6.8 to 16.3)	0.420
AJCC8 stage: II vs. I	0.1 (−5.6 to 5.8)	0.962	−0.6 (−7.3 to 6.2)	0.870
AJCC8 stage: III–IV vs. I	−1.6 (−9.9 to 6.7)	0.712	−0.2 (−8.7 to 8.3)	0.960
Time RT to survey (per year)	−0.1 (−0.8 to 0.6)	0.839	−0.5 (−1.3 to 0.3)	0.246
Prior surgery: yes vs. no	0.9 (−4.9 to 6.7)	0.759	−1.3 (−9.5 to 7.0)	0.763
Neck RT: unilateral vs. bilateral	−0.9 (−7.8 to 5.9)	0.788	−0.6 (−12.0 to 10.8)	0.917
QLQ‐HN43 swallowing (higher = worse)				
Age at RT (per 10 y)	2.7 (−0.6 to 6.1)	0.108	3.7 (0.2–7.2)	0.037
Smoking: former vs. never	5.2 (−0.4 to 10.8)	0.067	4.5 (−0.9 to 10.0)	0.100
Smoking: current vs. never	3.3 (−8.7 to 15.3)	0.589	1.2 (−10.0 to 12.4)	0.832
Chemotherapy: yes vs. no	6.6 (0.9–12.3)	0.024	3.6 (−3.6 to 10.9)	0.327
AJCC8 stage: II vs. I	7.1 (0.7–13.6)	0.030	4.4 (−2.3 to 11.1)	0.197
AJCC8 stage: III‐IV vs. I	9.7 (0.9–18.4)	0.031	4.7 (−4.8 to 14.2)	0.335
Time RT to survey (per year)	0.5 (−0.2 to 1.3)	0.140	0.8 (−0.0 to 1.6)	0.063
Prior surgery: yes vs. no	−8.3 (−13.3 to −3.2)	0.001	−4.0 (−10.8 to 2.8)	0.245
Neck RT: unilateral vs. bilateral	−6.9 (−13.0 to −0.9)	0.025	1.2 (−7.1 to 9.5)	0.777
QLQ‐HN35 Dry mouth and sticky saliva (higher = worse)				
Age at RT (per 10 y)	−1.1 (−6.2 to 4.0)	0.673	−0.9 (−6.6 to 4.7)	0.747
Smoking: former vs. never	5.0 (−4.0 to 14.1)	0.276	3.3 (−5.8 to 12.4)	0.474
Smoking: current vs. never	6.7 (−11.9 to 25.4)	0.480	3.8 (−14.4 to 22.1)	0.682
Chemotherapy: yes vs. no	5.9 (−4.7 to 16.5)	0.277	−4.1 (−15.2 to 7.0)	0.469
AJCC8 stage: II vs. I	10.7 (1.5–19.9)	0.022	7.3 (−2.8 to 17.4)	0.159
AJCC8 stage: III–IV vs. I	9.9 (−5.3 to 25.1)	0.200	5.3 (−11.6 to 22.3)	0.538
Time RT to survey (per year)	0.9 (−0.2 to 2.0)	0.122	0.5 (−0.9 to 1.8)	0.510
Prior surgery: yes vs. no	−7.7 (−17.6 to 2.2)	0.129	−0.5 (−13.3 to 12.4)	0.941
Neck RT: unilateral vs. bilateral	−16.3 (−26.7 to −5.9)	0.002	−14.0 (−27.6 to −0.5)	0.042
QLQ‐HN43 Speech (higher = worse)				
Age at RT (per 10 y)	−0.6 (−4.2 to 2.9)	0.733	1.2 (−2.7 to 5.0)	0.552
Smoking: former vs. never	0.1 (−6.0 to 6.3)	0.965	0.5 (−5.3 to 6.3)	0.865
Smoking: current vs. never	0.6 (−13.2 to 14.3)	0.937	−0.6 (−14.7 to 13.4)	0.928
Chemotherapy: yes vs. no	−2.9 (−10.5 to 4.7)	0.462	−5.0 (−14.4 to 4.5)	0.304
AJCC8 stage: II vs. I	5.6 (−1.4 to 12.7)	0.115	4.9 (−2.3 to 12.1)	0.186
AJCC8 stage: III–IV vs. I	3.0 (−5.6 to 11.6)	0.500	0.9 (−8.1 to 10.0)	0.840
Time RT to survey (per year)	0.9 (0.1–1.8)	0.025	1.0 (0.0–2.0)	0.043
Prior surgery: yes vs. no	−1.9 (−7.8 to 3.9)	0.519	−1.4 (−10.8 to 7.9)	0.763
Neck RT: unilateral vs. bilateral	−1.6 (−9.5 to 6.3)	0.689	0.1 (−11.6 to 11.8)	0.987
QLQ‐HN43 Pain in the mouth (higher = worse)				
Age at RT (per 10 y)	1.5 (−1.3 to 4.3)	0.303	1.6 (−1.5 to 4.6)	0.310
Smoking: former vs. never	2.1 (−2.8 to 7.0)	0.407	2.2 (−2.6 to 7.0)	0.363
Smoking: current vs. never	3.5 (−6.3 to 13.3)	0.487	2.5 (−7.7 to 12.6)	0.633
Chemotherapy: yes vs. no	−2.8 (−9.7 to 4.1)	0.429	−2.7 (−11.6 to 6.1)	0.544
AJCC8 stage: II vs. I	2.4 (−3.0 to 7.8)	0.380	3.0 (−3.0 to 9.0)	0.330
AJCC8 stage: III–IV vs. I	3.5 (−3.7 to 10.7)	0.340	3.5 (−4.7 to 11.6)	0.404
Time RT to survey (per year)	0.1 (−0.5 to 0.7)	0.692	0.3 (−0.5 to 1.0)	0.475
Prior surgery: yes vs. no	−3.4 (−7.9 to 1.0)	0.129	−4.6 (−10.8 to 1.7)	0.153
Neck RT: unilateral vs. bilateral	2.6 (−3.4 to 8.5)	0.398	6.1 (−3.4 to 15.5)	0.208

*Note: β* = adjusted mean difference in score (points). Reference categories: never smoker, no chemotherapy, AJCC 8th edition Stage I, no prior surgery, bilateral neck irradiation.

**TABLE 6:** Prespecified dosimetric predictors of continuous QOL outcomes (*n* = 186).

QOL outcome	Dosimetric parameter (predictor)	Unadjusted β (95% CI)	*p*	Adjusted *β* ^a^ (95% CI)	*p*	*q* (BH)
Dry mouth/sticky saliva	Contralateral parotid mean (per 10 Gy)	10.5 (5.2–15.9)	< 0.001	10.1 (3.7–16.5)	0.002	0.021
Dry mouth/sticky saliva	Ipsilateral parotid mean (per 10 Gy)	5.1 (1.8–8.5)	0.003	5.0 (0.4–9.6)	0.034	0.186
Dry mouth/sticky saliva	Oral cavity mean (per 10 Gy)	6.3 (1.7–10.8)	0.007	4.4 (−1.0 to 9.8)	0.111	0.306
Swallowing	Pharyngeal constrictors mean (per 10 Gy)	4.6 (1.6–7.7)	0.003	2.5 (−1.5 to 6.4)	0.219	0.411
Swallowing	Larynx mean (per 10 Gy)	2.4 (−1.3 to 6.2)	0.205	0.1 (−5.1 to 5.2)	0.982	0.982
Speech	Larynx mean (per 10 Gy)	0.4 (−2.6 to 3.4)	0.783	−2.3 (−5.9 to 1.4)	0.224	0.411
Speech	Oral cavity mean (per 10 Gy)	1.5 (−1.6 to 4.7)	0.345	−0.4 (−4.4 to 3.5)	0.827	0.982
Pain in the mouth	Oral cavity mean (per 10 Gy)	0.2 (−2.2 to 2.6)	0.855	−0.3 (−3.2 to 2.6)	0.859	0.982
Global Health Status	CTV high dose volume (per 10 cc)	−0.0 (−0.7 to 0.6)	0.921	−0.1 (−0.9 to 0.7)	0.893	0.982
Global Health Status	CTV intermediate dose volume (per 10 cc)	−0.5 (−1.1 to 0.2)	0.143	−0.6 (−1.2 to 0.0)	0.064	0.235
Global Health Status	CTV low dose volume (per 10 cc)	−0.3 (−0.8 to 0.2)	0.254	−0.3 (−0.8 to 0.3)	0.354	0.556

*Note: β* = change in QOL score per 10 Gy (mean dose parameters) or per 10 cc (CTV volumes). *q* (BH) = Benjamini‐Hochberg (BH) false‐discovery‐rate adjusted *p*‐value across all 11 comparisons.

^a^ Adjusted for age, smoking, stage, chemotherapy and time since radiotherapy.

## 4 Discussion

This large cross‐sectional study describes long‐term patient reported QOL after curative‐intent RT for OPSCC at an Australian tertiary cancer centre. This study reported excellent oncological outcomes in a real‐world setting, with favourable global health status and low levels of treatment regret. However, more than one quarter of patients reported worse health than before treatment and most of these respondents attributed the change to RT. Higher contralateral mean parotid dose was the only modifiable dosimetric predictor of poorer long‐term xerostomia, with unilateral neck irradiation associated with a clinically meaningful reduction in dry mouth and sticky saliva, highlighting potential areas for treatment refinement.

The reported global and HN specific QOL outcomes were broadly comparable to those previously reported in the literature. The median and mean global QOL scores were 83 (IQR 67–100) and 81 respectively, similar to reference head and neck cancer cohorts [20]. They were also more than 10 points higher (a clinically significant difference) than international general population reference values [21, 22]. However, this should be interpreted in light of the highly selected long‐term survivor cohort which may have overestimated QOL. Dry mouth/sticky saliva was the most affected HN specific domain, consistent with the EORTC HN‐43 validation study [19] and ARTFORCE III trial [20].

RT plans can be optimised in a variety of ways to reduce dose to critical OARs, but clinically meaningful benefit is most likely when RT plan optimisation is guided by validated relationships between OAR dosimetry and specific QOL endpoints. The PARSPORT trial demonstrated that parotid‐sparing IMRT (compared to non parotid‐sparing IMRT) was associated with reduced xerostomia and improved QLQ‐C30 Global and HN43 dry mouth QOL scores [8]. Consistent with this, the current study found each 10 Gy increase in contralateral parotid mean dose was associated with 10.1 point worsening in dry mouth/sticky saliva, which was a clinically meaningful correlation. The ipsilateral parotid association was weaker and did not hold true after FDR correction. One possible explanation is that the current cohort had significantly lower doses to the ipsilateral parotid (mean 29Gy compared to 47.6Gy) and contralateral parotid (mean 19Gy compared to 25.4Gy) than the parotid‐sparing IMRT arm of the PARSPORT trial [8] suggesting that even lower parotid doses are achievable.

Limitations of this study include the cross‐sectional design, absence of baseline QOL, and lack of comparable clinician‐reported toxicity. Because no baseline assessment was available, the reported symptom burden reflects patients' perceptions at a single post‐treatment time point and cannot distinguish effects attributable to RT from pre‐existing patient or disease‐related factors. As a retrospective study of all eligible patients, no a priori power calculation was performed; the cohort size was fixed by the available population, and confidence intervals are reported throughout so that the precision of each estimate is explicit. Analyses of less frequently affected domains, and of the small p16‐negative and prior‐surgery subgroups, should thus be interpreted with caution, particularly where confidence intervals are wide. Reported late QOL outcomes may be more favourable than the entire eligible cohort, as patients with more advanced disease upfront and/or those who received more intensive treatment (and thus expected to have worse long term QOL) might be less likely to complete questionnaires either due to disease relapse or death, or worse health which may prevent involvement in such studies. The survivorship bias (with only living patients) is likely to render the reported long‐term QOL more optimistic than that of the treated population as a whole. Survey completers were younger and more often had p16‐positive disease, earlier stage, and lower dose/volume treatment characteristics than non‐completers (Table S3). A related consequence is that p16 status and baseline performance status could not be evaluated as predictors of late QOL, since almost all survivors who responded were p16‐positive and ECOG 0. The findings therefore described a selected group of long‐term survivors and should be generalised with caution.

The survey design attempted to strike a pragmatic balance between length (known to affect completion rate) and completeness of QOL information obtained. However, the inclusion of swallowing specific instruments such as the MDADI (the primary outcome in de‐escalation trials [23, 24, 25]), measures of financial toxicity and loneliness, or dedicated swallowing‐, speech‐ and voice‐specific instruments would have been a useful addition. Socioeconomic factors, which have previously been demonstrated to influence QOL [26, 27], were also not well captured. Despite the acknowledged limitations, the strengths of this study include long follow up (median time to survey completion since RT greater than 5 years) and high survey completion rate (80%). The single centre design limits external validity but did allow for analysis in a cohort undergoing standardised RT treatment, supportive care, and follow up protocols per institutional guidelines.

The late QOL and oncologic outcomes reported here serve as an institutional baseline for patients treated with standard curative‐intent RT, with or without chemotherapy, before implementation of personalised or de‐escalated approaches. The hazard of death was 85% lower for patients with p16‐positive disease than those with p16‐negative disease (HR 0.15, 95% CI 0.09–0.25), and many of these patients may be considered for de‐escalation trials in the future, including our institution's currently accruing trial using FMISO PET to select patients with HPV‐OPSCC for radical RT dose de‐escalation [28]. Although inherently limited by aspects of the methodology, this study highlights important QOL issues for patients after RT for OPSCC, as well as potential contributing clinicopathologic and treatment variables which may warrant further investigation. The present results identify persistent QOL concerns and clinically relevant treatment factors for prospective validation.

## 5 Conclusions

Among long‐term OPSCC survivors treated with curative intent RT, with or without chemotherapy, global QOL was favourable, and treatment regret was rare, but dry mouth and sticky saliva remained a substantial late burden. Higher contralateral parotid mean dose was associated with worse dry mouth/sticky saliva; unilateral neck irradiation was also associated with lower symptom scores. These findings support continued prioritisation of contralateral parotid sparing and of unilateral irradiation where oncologically appropriate, while requiring confirmation in prospective longitudinal cohorts.

## Author Contributions

**Anna Lawless:** conceptualization, methodology, data curation, investigation, formal analysis, writing – original draft, writing – review and editing, project administration. **Dasantha Jayamanne:** conceptualization, methodology, supervision, writing – review and editing. **Lisa Parker:** investigation, writing – review and editing, data curation. **Esther Duruchukwu:** investigation, writing – review and editing, data curation. **Paula Macleod:** investigation, writing – review and editing. **Venkatesha Venkatesha:** formal analysis, writing – review and editing. **Christopher Brown:** formal analysis, writing – review and editing. **Alexander Guminski:** conceptualization, supervision, writing – review and editing. **Adrian Lee:** supervision, writing – review and editing. **Michael Back:** supervision, writing – review and editing. **Thomas Eade:** writing – review and editing, supervision. **Sarah Bergamin:** conceptualization, methodology, supervision, writing – review and editing.

## Funding

The authors have nothing to report.

## Ethics Statement

Ethics was approved by the Northern Sydney Local Health District Human Research Ethics Committee (Approval Number: 2023/ETH01944. Date: 3rd October 2023).

## Consent

Written informed consent was obtained from all participants prior to participation in the study.

## Conflicts of Interest

The authors declare no conflicts of interest.

## Supporting information


**Figure S1:** Quality of life survey distributed to participants.
**Figure S2:** Distribution and completion rates of quality‐of‐life survey.
**Table S1:** Head and neck specific quality of life survey responses (per each question of EORTC HN‐43).
**Table S2:** Missing data in the full cohort and among survey completers.
**Table S3:** Survey completers compared with non‐completers.

## Data Availability

The datasets generated and analysed during the current study are not publicly available due to ethical and privacy considerations involving patient data but are available from the corresponding author on reasonable request, subject to institutional and ethics committee approval.
