# Supplementary material for: Long‐Term Patient‐Reported Outcomes and Dosimetric Predictors of Quality of Life After Curative‐Intent Radiation Therapy for Oropharyngeal Squamous Cell Carcinoma: A Cross‐Sectional Study
Source: Cancer Rep (Hoboken). 2026 Oct 7;9(10):e70712. doi: 10.1002/cnr2.70712 (PMC13645483; doi:10.1002/cnr2.70712)
Supplement: Supplementary file 1 [file CNR2-9-e70712-s001.docx]

**Supplementary Tables & Figures**

Supplementary Figure 1. Quality of Life Survey Distributed to Participants

Supplementary Figure 2. Distribution and Completion Rates of Quality-of-Life Survey

Supplementary Table 1. Head and Neck Specific Quality of Life Survey Responses (per each question of EORTC HN-43)

Supplementary Table 2. Missing data in the full cohort and among survey completers

Supplementary Table 3. Survey completers compared with non-completers

Supplementary Figure 1. Quality of Life Survey Distributed to Participants

Supplementary Figure 2. Distribution and Completion Rates of Quality-of-Life Survey

**The 13 patients not contacted for ‘other reasons’ included known relocation interstate/overseas or into care facility without available contact details and patients flagged by clinical team as having significant comorbidities and/or care needs precluding study involvement.*

Supplementary Table 1. Head and Neck Specific Quality of Life Survey Responses (per each question of EORTC HN-43)

| **Variable** | **Response** | | | |
| --- | --- | --- | --- | --- |
|  | **Not at All** | **A Little** | **Quite a Bit** | **Very Much** |
| Pain in your mouth? | 136 (73%) | 40 (22%) | 9 (4.8%) | 1 (0.5%) |
| Pain in your jaw? | 137 (74%) | 37 (20%) | 11 (5.9%) | 1 (0.5%) |
| Soreness in your mouth? | 131 (70%) | 42 (23%) | 11 (5.9%) | 2 (1.1%) |
| Pain in your throat? | 117 (63%) | 58 (31%) | 10 (5.4%) | 1 (0.5%) |
| Problems swallowing liquids? | 121 (65%) | 53 (28%) | 9 (4.8%) | 3 (1.6%) |
| Problems swallowing pureed food? | 154 (83%) | 26 (14%) | 4 (2.2%) | 2 (1.1%) |
| Problems swallowing solid food? | 58 (31%) | 86 (46%) | 25 (13%) | 17 (9.1%) |
| Have you choked when swallowing? | 96 (52%) | 70 (38%) | 13 (7.0%) | 7 (3.8%) |
| Problems with your teeth? | 90 (48%) | 42 (23%) | 28 (15%) | 26 (14%) |
| Problems because of losing some teeth? | 128 (69%) | 20 (11%) | 17 (9.1%) | 21 (11%) |
| Problems opening your mouth wide? | 108 (58%) | 52 (28%) | 16 (8.6%) | 10 (5.4%) |
| Dry mouth? | 32 (17%) | 62 (33%) | 54 (29%) | 38 (20%) |
| Sticky saliva? | 76 (41%) | 56 (30%) | 35 (19%) | 19 (10%) |
| Problems with your sense of smell? | 117 (63%) | 54 (29%) | 11 (5.9%) | 4 (2.2%) |
| Problems with your sense of taste? | 77 (41%) | 64 (34%) | 31 (17%) | 14 (7.5%) |
| Problems with coughing? | 107 (58%) | 53 (28%) | 22 (12%) | 4 (2.2%) |
| Problems with hoarseness? | 99 (53%) | 68 (37%) | 16 (8.6%) | 3 (1.6%) |
| Problems with your appearance? | 144 (77%) | 30 (16%) | 5 (2.7%) | 7 (3.8%) |
| Felt less physically attractive RT? | 141 (76%) | 27 (15%) | 9 (4.8%) | 9 (4.8%) |
| Felt dissatisfied with your body? | 126 (68%) | 41 (22%) | 13 (7.0%) | 6 (3.2%) |
| Problems eating? | 79 (42%) | 73 (39%) | 15 (8.1%) | 19 (10%) |
| Problems eating in front of your family? | 141 (76%) | 31 (17%) | 6 (3.2%) | 8 (4.3%) |
| Problems eating in front of other people? | 126 (68%) | 37 (20%) | 10 (5.4%) | 13 (7.0%) |
| Problems enjoying your meals? | 100 (54%) | 53 (28%) | 16 (8.6%) | 17 (9.1%) |
| Problems talking to other people? | 141 (76%) | 31 (17%) | 11 (5.9%) | 3 (1.6%) |
| Problems talking on the telephone? | 144 (77%) | 29 (16%) | 11 (5.9%) | 2 (1.1%) |
| Problems talking in a noisy environment? | 100 (54%) | 54 (29%) | 23 (12%) | 9 (4.8%) |
| Problems speaking clearly? | 127 (68%) | 46 (25%) | 6 (3.2%) | 7 (3.8%) |
| Problems going out in public? | 165 (89%) | 13 (7.0%) | 6 (3.2%) | 2 (1.1%) |
| Felt less interest in sex? | 114 (61%) | 49 (26%) | 13 (7.0%) | 10 (5.4%) |
| Felt less sexual enjoyment? | 116 (62%) | 45 (24%) | 12 (6.5%) | 13 (7.0%) |
| Problems raising/moving your arm? | 149 (80%) | 26 (14%) | 6 (3.2%) | 5 (2.7%) |
| Pain in your shoulder? | 123 (66%) | 40 (22%) | 20 (11%) | 3 (1.6%) |
| Swelling in your neck? | 140 (75%) | 36 (19%) | 6 (3.2%) | 4 (2.2%) |
| Skin problems (e.g. itchy, dry)? | 112 (60%) | 63 (34%) | 8 (4.3%) | 3 (1.6%) |
| Had a rash? | 154 (83%) | 28 (15%) | 4 (2.2%) | 0 (0%) |
| Skin changed colour? | 152 (82%) | 26 (14%) | 8 (4.3%) | 0 (0%) |
| Worried that your weight is too low? | 146 (78%) | 26 (14%) | 11 (5.9%) | 3 (1.6%) |
| Worried about the results of examinations and tests? | 112 (60%) | 59 (32%) | 11 (5.9%) | 4 (2.2%) |
| Worried about your health in the future? | 62 (33%) | 92 (49%) | 24 (13%) | 8 (4.3%) |
| Problems with wounds healing? | 149 (80%) | 28 (15%) | 6 (3.2%) | 3 (1.6%) |
| Tingling or numbness in your hands or feet? | 105 (56%) | 54 (29%) | 16 (8.6%) | 11 (5.9%) |
| Problems chewing? | 103 (55%) | 53 (28%) | 18 (9.7%) | 12 (6.5%) |

Supplementary Table 2. Missing data in the full cohort and among survey completers

| **Variable** | **Missing, full cohort (n = 344)** | **%** | **Missing, completers (n = 186)** | **%** |
| --- | --- | --- | --- | --- |
| p16 | 44 | 12.8% | 15 | 8.1% |
| ECOG | 8 | 2.3% | 4 | 2.2% |
| Smoking | 1 | 0.3% | 0 | 0.0% |
| AJCC 8th stage | 0 | 0.0% | 0 | 0.0% |
| Chemotherapy | 0 | 0.0% | 0 | 0.0% |
| Prior surgery | 0 | 0.0% | 0 | 0.0% |
| Neck RT laterality | 3 | 0.9% | 0 | 0.0% |
| CTV HD vol | 24 | 7.0% | 0 | 0.0% |
| CTV ID vol | 26 | 7.6% | 0 | 0.0% |
| CTV LD vol | 24 | 7.0% | 0 | 0.0% |
| Parotid ipsi | 26 | 7.6% | 0 | 0.0% |
| Parotid contra | 24 | 7.0% | 0 | 0.0% |
| Oral cavity mean | 128 | 37.2% | 0 | 0.0% |
| Pharyngeal constrictors mean | 151 | 43.9% | 0 | 0.0% |
| Larynx mean | 29 | 8.4% | 0 | 0.0% |

*Note: Among the 186 completers there is no missing data on any questionnaire item, model covariate or dosimetric parameter, so the QOL analyses are complete case only in a nominal sense. In the full cohort the notable gaps are p16 status (44 untested) and pharyngeal constrictor and oral cavity contours, which were extracted only for the surveyed subset.*

Supplementary Table 3. Survey completers compared with non-completers (full cohort, n = 344)

| **Variable** | **Completers (n = 186)** | **Non-completers (n = 158)** | **SMD** | **p** |
| --- | --- | --- | --- | --- |
| Age at RT, mean (SD) | 60.3 (9.2) | 62.8 (9.9) | -0.26 | 0.036 |
| Sex: Female | 20 (11%) | 26 (16%) |  | 0.165 |
| Sex: Male | 166 (89%) | 132 (84%) |  | 0.165 |
| p16: Negative | 3 (2%) | 23 (15%) |  | <0.001 |
| p16: Not tested | 15 (8%) | 29 (18%) |  | <0.001 |
| p16: Positive | 168 (90%) | 106 (67%) |  | <0.001 |
| Smoking: Current | 15 (8%) | 41 (26%) |  | <0.001 |
| Smoking: Former | 92 (49%) | 68 (43%) |  | <0.001 |
| Smoking: Never | 79 (42%) | 48 (31%) |  | <0.001 |
| AJCC 8th stage: I | 100 (54%) | 41 (26%) |  | <0.001 |
| AJCC 8th stage: II | 59 (32%) | 59 (37%) |  | <0.001 |
| AJCC 8th stage: III-IV | 27 (15%) | 58 (37%) |  | <0.001 |
| Chemotherapy: No | 32 (17%) | 22 (14%) |  | 0.494 |
| Chemotherapy: Yes | 154 (83%) | 136 (86%) |  | 0.494 |
| Prior surgery: No | 142 (76%) | 142 (90%) |  | 0.002 |
| Prior surgery: Yes | 44 (24%) | 16 (10%) |  | 0.002 |
| Neck RT: Bilateral | 150 (81%) | 135 (87%) |  | 0.146 |
| Neck RT: Unilateral | 36 (19%) | 20 (13%) |  | 0.146 |
| CTV HD vol, mean (SD) | 69.1 (41.6) | 73.3 (48.2) | -0.09 | 0.527 |
| CTV ID vol, mean (SD) | 78.0 (49.8) | 90.3 (49.3) | -0.25 | 0.010 |
| CTV LD vol, mean (SD) | 132.1 (60.8) | 167.4 (88.3) | -0.47 | <0.001 |
| Parotid ipsi mean, mean (SD) | 31.4 (12.3) | 35.4 (11.4) | -0.34 | 0.003 |
| Parotid contra mean, mean (SD) | 17.6 (8.9) | 20.3 (8.8) | -0.31 | 0.004 |
| Oral cavity mean, mean (SD) | 45.5 (9.5) | 44.2 (10.1) | 0.14 | 0.242 |
| Pharyngeal constrictors mean, mean (SD) | 53.2 (8.5) | 46.2 (13.9) | 0.6 | 0.208 |
| Larynx mean, mean (SD) | 30.3 (9.1) | 32.1 (8.1) | -0.2 | 0.015 |

*SMD = standardised mean difference. Continuous variables compared with the Mann-Whitney U test, categorical variables with the chi-square or Fisher exact test.*
